# Stable-Isotope-Informed, Genome-Resolved Metagenomics Uncovers Potential Cross-Kingdom Interactions in Rhizosphere Soil

**DOI:** 10.1128/mSphere.00085-21

**Published:** 2021-09-01

**Authors:** Evan P. Starr, Shengjing Shi, Steven J. Blazewicz, Benjamin J. Koch, Alexander J. Probst, Bruce A. Hungate, Jennifer Pett-Ridge, Mary K. Firestone, Jillian F. Banfield

**Affiliations:** a Department of Plant and Microbial Biology, University of California, Berkeley, California, USA; b Lincoln Science Centre, AgResearch Ltd., Christchurch, New Zealand; c Physical and Life Sciences Directorate, Lawrence Livermore National Laboratory, Livermore, California, USA; d Department of Biological Sciences, Northern Arizona University, Flagstaff, Arizona, USA; e Center for Ecosystem Science and Society, Northern Arizona University, Flagstaff, Arizona, USA; f Biofilm Center, University of Duisburg—Essen, Essen, Germany; g Department of Environmental Science, Policy, and Management, University of California, Berkeley, California, USA; h Earth and Environmental Sciences, Lawrence Berkeley National Laboratory, Berkeley, California, USA; i Department of Earth and Planetary Science, University of California, Berkeley, California, USA; j Innovative Genomics Institute, Berkeley, California, USA; k Chan Zuckerberg Biohub, San Francisco, California, USA; University of Michigan—Ann Arbor

**Keywords:** bacteriophages, metagenomics, plant-microbe interactions, rhizosphere, stable-isotope probing

## Abstract

The functioning, health, and productivity of soil are intimately tied to a complex network of interactions, particularly in plant root-associated rhizosphere soil. We conducted a stable-isotope-informed, genome-resolved metagenomic study to trace carbon from Avena fatua grown in a ^13^CO_2_ atmosphere into soil. We collected paired rhizosphere and nonrhizosphere soil at 6 and 9 weeks of plant growth and extracted DNA that was then separated by density using ultracentrifugation. Thirty-two fractions from each of five samples were grouped by density, sequenced, assembled, and binned to generate 55 unique bacterial genomes that were ≥70% complete. We also identified complete 18S rRNA sequences of several ^13^C-enriched microeukaryotic bacterivores and fungi. We generated 10 circularized bacteriophage (phage) genomes, some of which were the most labeled entities in the rhizosphere, suggesting that phage may be important agents of turnover of plant-derived C in soil. CRISPR locus targeting connected one of these phage to a *Burkholderiales* host predicted to be a plant pathogen. Another highly labeled phage is predicted to replicate in a *Catenulispora* sp., a possible plant growth-promoting bacterium. We searched the genome bins for traits known to be used in interactions involving bacteria, microeukaryotes, and plant roots and found DNA from heavily ^13^C-labeled bacterial genes thought to be involved in modulating plant signaling hormones, plant pathogenicity, and defense against microeukaryote grazing. Stable-isotope-informed, genome-resolved metagenomics indicated that phage can be important agents of turnover of plant-derived carbon in soil.

**IMPORTANCE** Plants grow in intimate association with soil microbial communities; these microbes can facilitate the availability of essential resources to plants. Thus, plant productivity commonly depends on interactions with rhizosphere bacteria, viruses, and eukaryotes. Our work is significant because we identified the organisms that took up plant-derived organic C in rhizosphere soil and determined that many of the active bacteria are plant pathogens or can impact plant growth via hormone modulation. Further, by showing that bacteriophage accumulate CO_2_-derived carbon, we demonstrated their vital roles in redistribution of plant-derived C into the soil environment through bacterial cell lysis. The use of stable-isotope probing (SIP) to identify consumption (or lack thereof) of root-derived C by key microbial community members within highly complex microbial communities opens the way for assessing manipulations of bacteria and phage with potentially beneficial and detrimental traits, ultimately providing a path to improved plant health and soil carbon storage.

## INTRODUCTION

Plant-derived carbon provides the energetic basis for an intricate web of life in soil, among the world’s most complex microbial ecosystems. Many heterotrophic soil organisms are sustained primarily by the carbon that is fixed by plants and released into soil in the region surrounding growing roots, the rhizosphere. Root-derived carbon not only supports a bloom of microbial activity and biomass growth but also stimulates microbial interactions that play roles in plant immunity and nutrient acquisition and lead to organic matter associations with soil minerals (1).

The soil ecosystem is characterized by interactions occurring among organisms across trophic levels, which may direct the fate of plant-derived carbon in soil. These interactions can be difficult to investigate because of the tremendous physical and chemical heterogeneity of soil and resulting vast biological diversity. Much of the recent work on soil microbiology has been sequence based and focused on generating inventories of bacteria and archaea using 16S rRNA gene fragments (2, 3) or fungi using internal transcribed spacer region sequencing (4, 5). However, since DNA extracted from soil includes genes from virtually all organisms present, it is possible to use shotgun metagenome sequencing to profile complete soil communities, potentially with genomic resolution. This is important because genomes provide not only phylogenetic information but also a cache of functional predictions. However, only recently have studies achieved genomic resolution in soil, largely due to strain complexity and relatively even abundance levels (6–10). Bacterial genomes possess a huge variety of known and unknown genes, including those that comprise biosynthetic gene clusters (BGCs). Such biosynthetic pathways hold technological relevance but are also fundamental for soil ecology, as their products could mediate interorganismal interactions, including antagonistic interactions via antibiotics, mineral interactions via siderophores, and signaling compounds (11, 12). While there has been a surge of interest in viral diversity and ecology, there have been relatively few studies documenting bacterium-phage interactions in soil (13–15). Also present in soil are fungi, protists, and larger organisms that have been documented via 18S rRNA gene sequencing (16, 17) and more classical methods (18, 19). While some studies have documented bipartite interactions in soil, such as fungi and bacteria (20, 21), bacteria and phage (13, 22), and plant and fungi (23, 24), detailing complex cross-kingdom interactions in soil remains a huge challenge.

Stable-isotope probing (SIP) provides one means of tracing elemental flow among soil community members. SIP studies have been conducted in a variety of ecosystems, including hot springs and the animal gut, using a range of isotopes and labeled substrates (25–28). SIP techniques can use carbon-fixing organisms to generate biomass and complex mixes of compounds to investigate general processes such as decomposition of litter or C flow in the rhizosphere (29–33). Stable isotopes can also be monitored between trophic levels, allowing the study of microbial predation and phage activity (34, 35). A recently developed variant, quantitative SIP (qSIP), makes these measurements possible at the individual or population genome scale (36). This is facilitated by comparing the taxon-specific density for each sequenced entity in labeled and unlabeled DNA fractions (36, 37). From qSIP data, it is possible to estimate the gross growth rate of organisms assimilating labeled carbon substrates.

To better understand the movement of carbon introduced into soil as plant-derived rhizodeposits, we combined stable-isotope probing and genome-resolved metagenomics. We grew common wild oat grass, Avena fatua, for 6 and 9 weeks in a ^13^CO_2_-enriched atmosphere and tracked the isotopically enriched plant carbon as it was released into the surrounding soil community via exudation, decay of root biomass, and direct biotic transfers via root pathogen attack. We hypothesized that plant-derived carbon would be detected in multiple trophic levels and that genome analysis would provide clues as to the ecological means of this transfer. By separating extracted DNA based on density, we determined which populations consumed the isotopically heavy plant-derived carbon (or predated upon other organisms who were primary consumers) and incorporated it into their genomes during replication. Conversely, we also identified microorganisms present in substantial number that did not consume ^13^C-labeled root C. We used genome assembly and binning to analyze the genes at the organismal level. We chose to focus on binned bacterial genomes rather than studying all assembled scaffolds, as this allowed us to more accurately calculate population-level stable-isotope enrichment and interpret the functional significance of individual populations. In the metagenome-assembled genomes, we identified possible interaction signatures—such as genes for the production of plant hormones and modulation of hormone concentrations, secretion systems, and secondary metabolites. Assembled genomes also allowed us to predict whether an organism is a plant growth-promoting bacterium (PGPB) or a pathogen (38, 39).

## RESULTS

### ^13^CO_2_ plant labeling and stable-isotope probing.

A. fatua plants were grown in a continuously regenerated ^13^CO_2_ plant growth chamber. After 6 weeks of growth, the plant shoots were highly ^13^C labeled (∼94 atom%) (40, 41). DNA was extracted from bulk soil samples collected at the beginning of the study, 6 and 9 weeks, and also from paired rhizosphere soil from plants grown for 6 and 9 weeks (see Fig. S1 in the supplemental material), and then all DNA was fractionated in a density gradient (37). We compared the density separation of rhizosphere DNA to bulk soil DNA to identify unenriched DNA (light), a mixture of enriched and unenriched DNA (middle), and highly ^13^C enriched (heavy); these fractions were then shotgun sequenced (see Fig. S2 and Table S1 in the supplemental material). After assembly of the individual metagenomes, the heavier fractions (middle and heavy) had overall larger assemblies with a greater percentage of reads aligning to the assembly than the light fraction assemblies (Table S1).

10.1128/mSphere.00085-21.1 FIG S1 Experimental design of soil microcosms, stable-isotope probing density fractionation, and samples generated for sequencing from a ^13^CO_2_ labeling study of common wild oat, *Avena fatua*. Within the microcosms, hashed squares indicate root-excluding bulk soil bags. DNA fractionation plots show DNA density profiles from 0, 6, or 9 weeks of plant growth with unlabeled, partially labeled, and labeled community DNA indicated by gray, yellow, and peach shading, respectively. Brown lines and text indicate bulk soil samples, and green lines and text indicate rhizosphere samples. Download FIG S1, TIF file, 1.5 MB. Copyright © 2021 Starr et al. 2021 Starr et al. https://creativecommons.org/licenses/by/4.0/ This content is distributed under the terms of the Creative Commons Attribution 4.0 International license.

10.1128/mSphere.00085-21.2 FIG S2 DNA density profiles from a ^13^CO_2_ plant labeling study illustrate stable-isotope separation in DNA ^13^C enrichment between labeled (rhizosphere) and unlabeled (bulk soil) treatments. The density (gram/milliliter) and concentration (nanogram/microliter) of DNA for each collected fraction from each sample is plotted to generate these SIP separation graphs. Fractions were subsequently combined into “light,” “middle,” and “heavy” bins prior to sequencing. Download FIG S2, TIF file, 2.8 MB. Copyright © 2021 Starr et al. 2021 Starr et al. https://creativecommons.org/licenses/by/4.0/ This content is distributed under the terms of the Creative Commons Attribution 4.0 International license.

10.1128/mSphere.00085-21.9 TABLE S1 SIP, sequencing, and metagenome assembly statistics. Download Table S1, PDF file, 0.03 MB. Copyright © 2021 Starr et al. 2021 Starr et al. https://creativecommons.org/licenses/by/4.0/ This content is distributed under the terms of the Creative Commons Attribution 4.0 International license.

### Organisms identified from SIP metagenomes.

**FIG 1:**
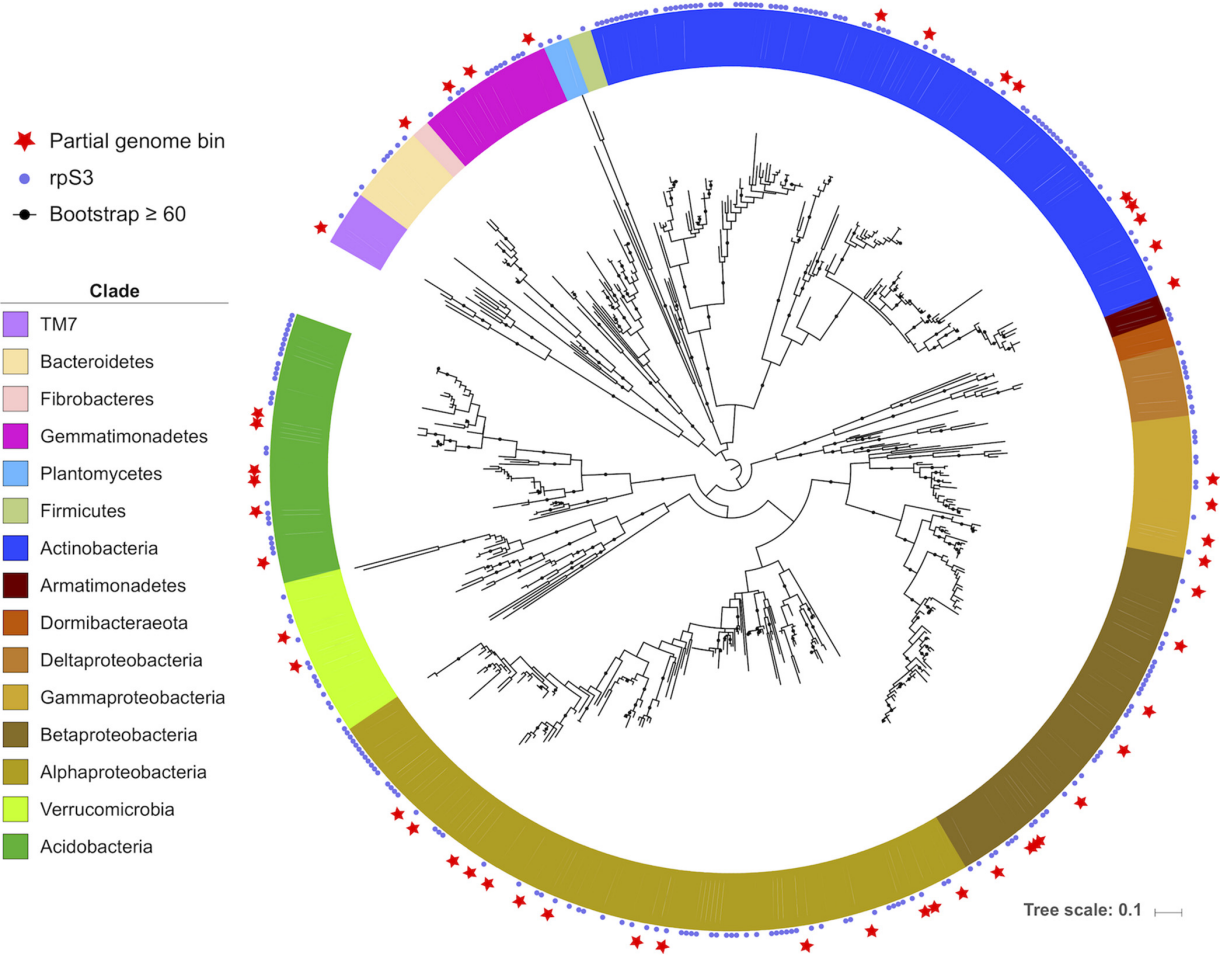
Phylogenetic tree illustrating the bacterial rpS3 genes identified in this study in the context of published rpS3 sequences. Soil- and rhizosphere-derived metagenomic bacterial bins (55 bins; >70% complete) with an rpS3 gene and unbinned scaffolds are marked (314 total). Publicly available representative species to provide phylogenetic grounding are also included. Bacterial clades are highlighted in different colors.

It is currently unrealistic to bin genomes for all organisms present in a soil sample, yet relatively extensive reconstruction of genome fragments is possible. We used an assembled marker gene approach to approximate microbial community composition, using the ribosomal protein S3 gene (rpS3), which is found in single copy on bacterial and archaeal genomes and has been used to profile microbial communities for phylogeny and abundance (42). The rpS3 gene tends to assemble well from metagenomes, and since it occurs as a single copy per genome, it provides a better abundance metric than 16S rRNA (42, 43). From each sample, we identified the rpS3 and dereplicated the sequences to a level of 99% nucleic acid identity (44). The resulting 314 distinct rpS3 sequences we identified represent a diverse array of soil bacteria (Fig. 1).

**FIG 2:**
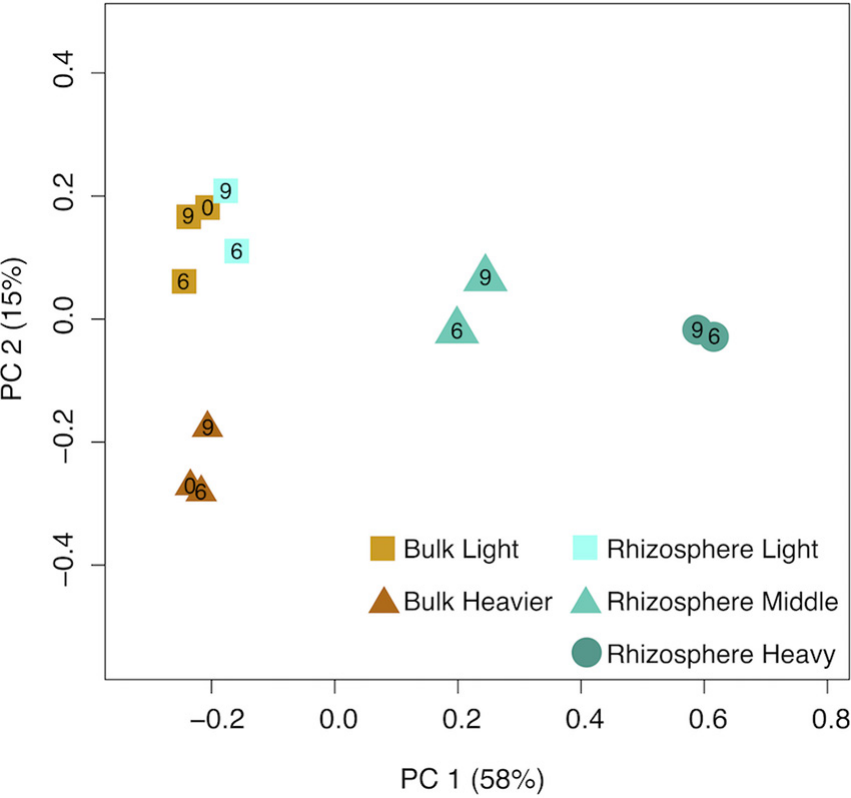
PCoA of bacterial rpS3 gene sequences from DNA from five soil samples fractionated into 12 SIP fractions collected from a ^13^CO_2_ plant labeling study. Symbols and colors represent the different samples and fractions. Numbers inside the symbols correspond to the week of sampling. See Fig. S1 in the supplemental material for additional explanation.

To study community dynamics across samples and fractions, we mapped the reads from each sample and fraction to scaffolds containing the rpS3 gene. We used the coverage of the scaffolds as a proxy for each organism’s relative abundance. Based on a principal coordinate analysis (PCoA), the fractions and samples show a clear community separation based on soil habitat (bulk versus rhizosphere) and SIP density fraction, but not time (Fig. 2). It is widely documented that density of DNA is affected both by its GC content (sequences with higher GC content are more dense than those with lower GC) and the enrichment level of the DNA (45). Because these two factors control DNA density, the bulk samples (even with no added ^13^C) separate into a lower GC light fraction cluster and a higher GC heavier fraction cluster. The bulk light fraction and rhizosphere light fraction samples group together. The rhizosphere middle fraction separates from both types of light fractions. The rhizosphere middle fraction also separates from the bulk soil heavier fraction, as it contains DNA from the high-GC organisms that did not incorporate the label and the community of lower-GC organisms that were labeled sufficiently to increase the density of their DNA. Finally, the rhizosphere heavy fractions separate from all other fractions—the bacteria present in this sample are almost entirely high-GC organisms that incorporated ^13^C into their DNA.

**FIG 3:**
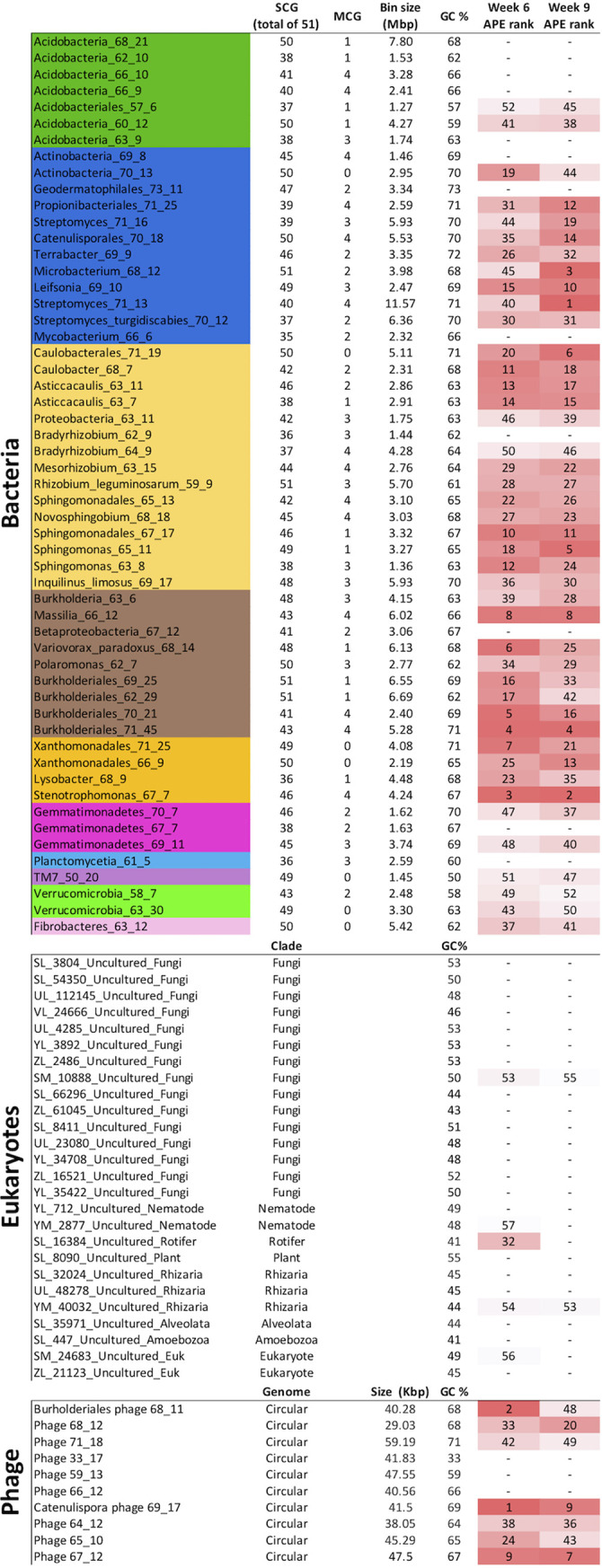
Genome and ^13^C isotope labeling statistics for metagenome-assembled bacterial genomes (colored by clade following the color scheme from Fig. 1), eukaryote scaffolds containing 18S rRNA genes, and complete phage genomes. Rank atom percent excess (APE) values (highlighted with red heat maps for each column) are derived from qSIP calculations. Bin completeness and contamination are presented as the number of 51 single copy genes (SCG) and number of multicopy genes (MCG).

From our 12 metagenomes (Fig. S1), we reconstructed and binned 55 bacterial genomes that were ≥70% complete with ≤10% contamination, as measured by the inventory of 51 single copy genes from ggKbase (Fig. 3 and Fig. S3). In addition to bacteria, we also detected a number of eukaryotes and phage. We reconstructed 27 complete 18S rRNA gene sequences from soil eukaryotes (Fig. 3 and Fig. S4). The soil microeukaryotes fall into a variety of soil clades, including Amoebozoa, Fungi, Metazoa (nematodes and rotifers), Rhizaria, and Alveolata. We also identified phage-derived DNA in our samples and reconstructed 10 complete, circularized phage genomes (Fig. 3).

10.1128/mSphere.00085-21.3 FIG S3 Count of bacterial single copy genes in partial genome bins identified in this study. Color bar represents the clade of bin, colored according to the legend presented in Fig. 1. Download FIG S3, TIF file, 2.8 MB. Copyright © 2021 Starr et al. 2021 Starr et al. https://creativecommons.org/licenses/by/4.0/ This content is distributed under the terms of the Creative Commons Attribution 4.0 International license.

10.1128/mSphere.00085-21.4 FIG S4 18S rRNA tree colored by clade. Black dots represent bootstrap values of ≥60. Long leaves were removed for ease of visualization, and 18S rRNA sequences identified in this study are shown in purple. Download FIG S4, TIF file, 2.8 MB. Copyright © 2021 Starr et al. 2021 Starr et al. https://creativecommons.org/licenses/by/4.0/ This content is distributed under the terms of the Creative Commons Attribution 4.0 International license.

**FIG 4:**
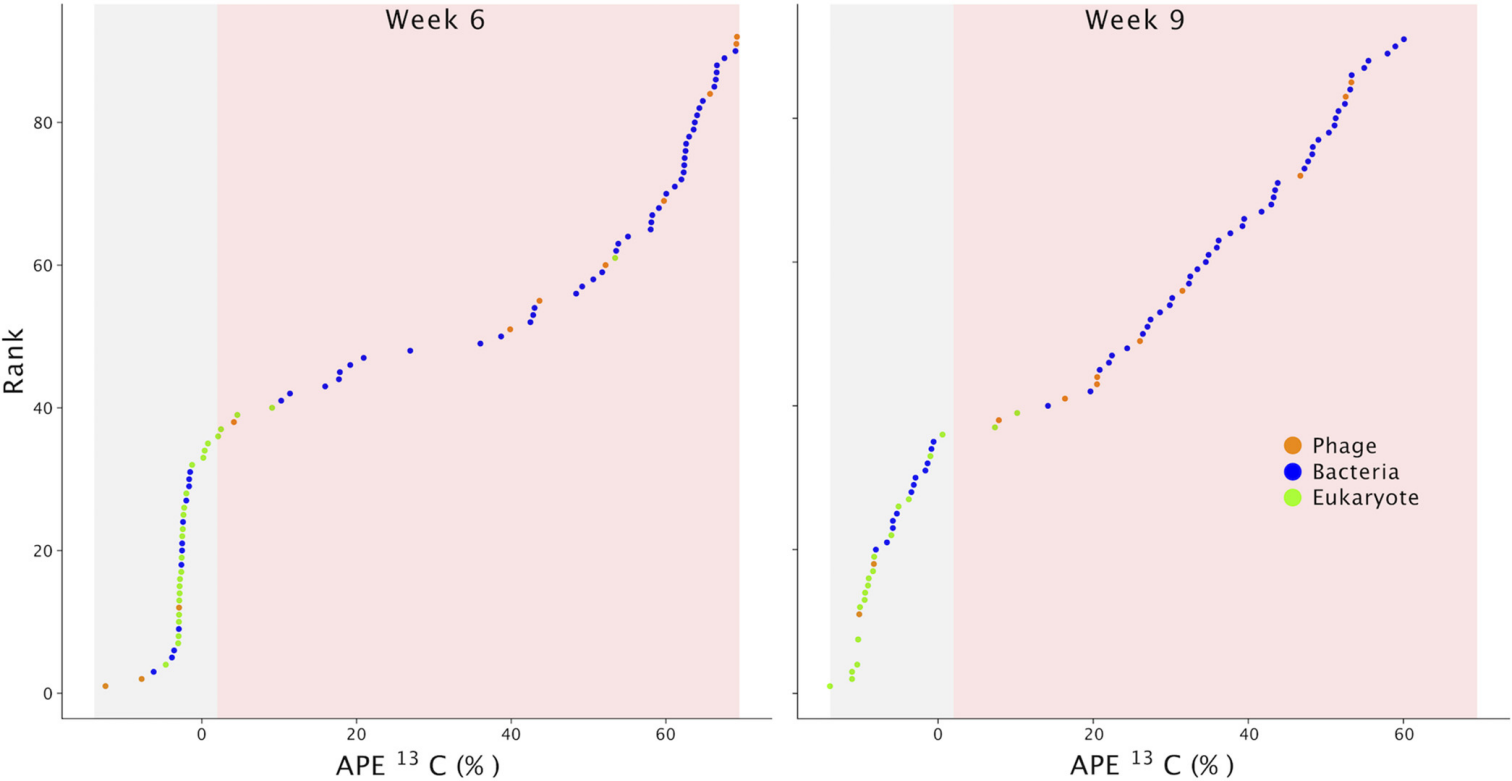
The rank of soil-derived phage genomes, bacterial genome bins, and scaffolds encoding eukaryotic 18S rRNA genes in week 6 and 9 in order of their atom percent excess (APE) based on the qSIP calculations. The gray region indicates unlabeled entities, and the pink region indicates predicted labeled DNA. The labeling cutoff is explained in Materials and Methods.

We used quantitative stable-isotope probing (qSIP) to estimate ^13^C atom percent excess (APE) for each taxon (36, 37). The qSIP method relies on tracking the shift in density, calculated by coverage in the different fractions, of a genome between unlabeled (bulk) and labeled (rhizosphere) samples, this metric is not influenced by the genome size. We mapped the reads of all samples against our dereplicated suite of 55 genome bins. We used the coverage of the scaffolds containing the 18S rRNA as a proxy for eukaryotic genome coverage because we were unable to bin eukaryotic genomes. Because of the lack of replicates and small number of fractions, we chose a conservative detection cutoff of 2.5% APE (see Materials and Methods); that is, any entities with an APE higher than this cutoff were interpreted as having detectably incorporated the ^13^C tracer (Fig. 4). We report the rank in order of enrichment (APE) for each sequence in Fig. 3. Many of the phage we identified appear to be highly labeled. Bacteria were also highly labeled, and a few of the eukaryote sequences were labeled, although to a much lesser degree than the bacterial genomes (Fig. 4). Of the 55 bacterial genomes we assembled, the majority (78%) were detectably ^13^C enriched.

We calculated the gross growth rate for each taxon on labeled carbon. The gross growth rate was calculated by assuming linear growth and comparing the abundance of each taxon at week 0 to the other time points and incorporating the mass of the DNA extracted and the mass of DNA from the original soil sample (36). The gross growth rate is presented in the unit: mass (in nanograms) of DNA per day per gram of dry soil. These taxon-specific growth rate estimates can be thought of as a measure of which individual populations in the rhizosphere grew on ^13^C-labeled plant-derived carbon. The gross growth rates indicated bacterial and phage growth but very limited eukaryotic growth on plant-derived carbon. Indeed, only a few specific bacteria and phage had high gross growth rates on plant derived-carbon (Fig. S5).

10.1128/mSphere.00085-21.5 FIG S5 Plots of atom percent excess (APR) versus gross growth rates of soil organisms (nanogram of DNA gram of soil^−1^ day^−1^) based on root-derived carbon. (Top) The log of gross growth per day on plant-derived carbon compared to APE, and (Bottom) violin plot of gross growth of different types of soil populations. Download FIG S5, TIF file, 2.8 MB. Copyright © 2021 Starr et al. 2021 Starr et al. https://creativecommons.org/licenses/by/4.0/ This content is distributed under the terms of the Creative Commons Attribution 4.0 International license.

### Plant-soil community interactions.

**FIG 5:**
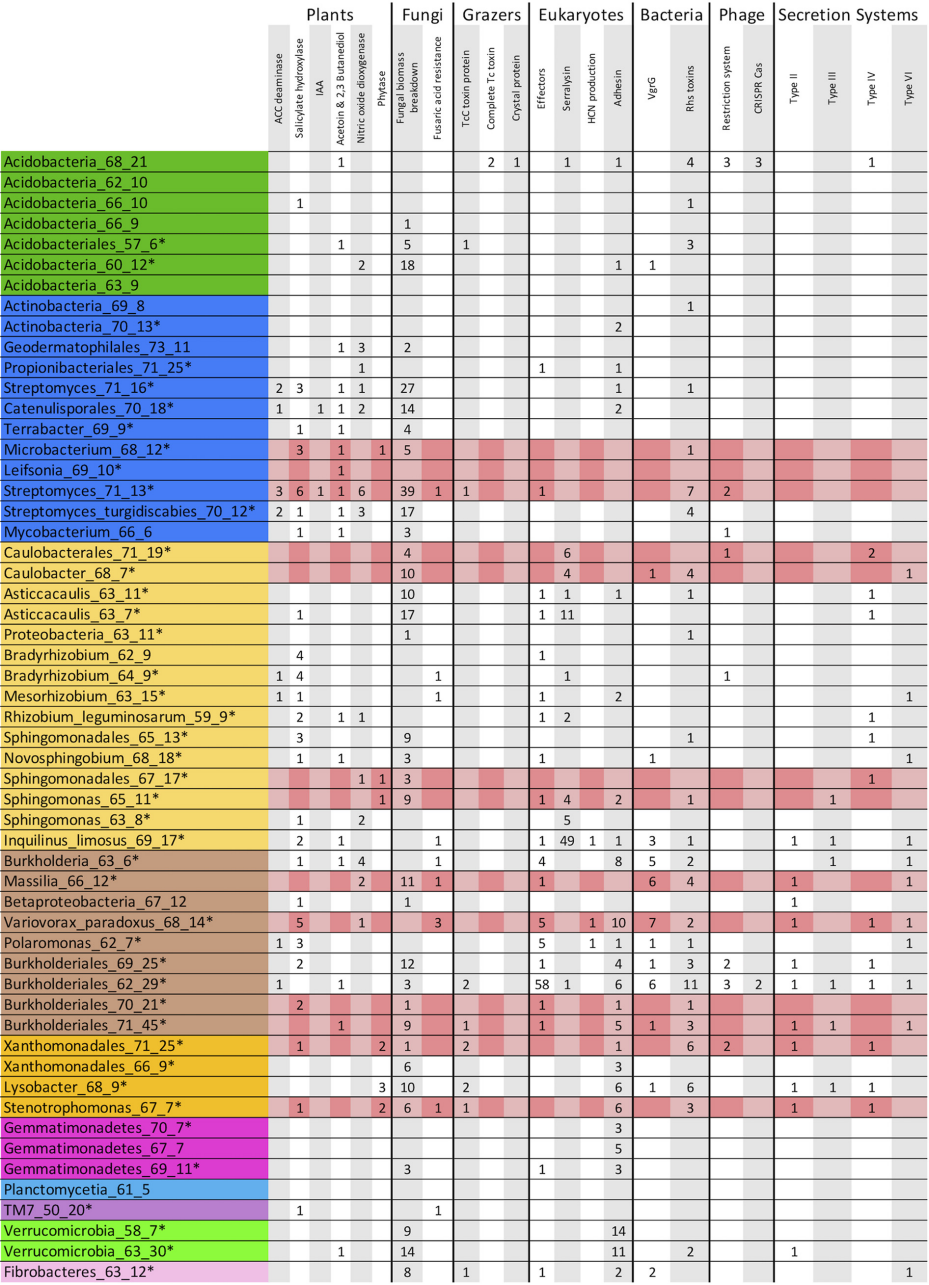
Possible interorganismal interactions encoded by 55 bacterial metagenome-assembled bacterial genomes identified in this study. Numbers indicate the number of individual genes or nearly complete pathways predicted to be used in interorganismal interaction. Asterisks signify genome bins with detectable ^13^C label, and pink highlighting indicates taxa that are among the top 15% of ^13^C-enriched populations.

Many of the bacterial genomes carrying plant interaction-mediating genes and pathways were also highly labeled, suggesting an intimate relationship between the plant roots and growing bacteria (Fig. 5). Bacteria that are closely associated with plants may degrade plant infection signaling hormones to avoid detection during plant colonization. Many of the bacterial genomes we binned carry genes that encode the ability to hydrolyze salicylic acid (Fig. 5), a common phenolic plant hormone used in pathogen defense signaling (46). However, this protein can also be used to degrade other phenolic compounds (47). Thus, we examined the nearby genomic regions for clues about the function of the gene. In one instance, the salicylate hydroxylase gene in the Microbacterium_68_12 genome is surrounded by a variety of glycosyl hydrolases and esterases that act on plant cell wall polymers, indicating that this region of the genome may be devoted to plant cell invasion and avoidance of detection. Additionally, many of the rhizosphere-dwelling populations that we detected carry genes that encode the ability to degrade nitric oxide gas (Fig. 5), another pathogen defense hormone (48).

Although plant-derived carbon is the main source of ^13^C used by the soil community, other organisms may be capable of fixing CO_2_. However, we found no evidence for carbon fixation pathways in the bacterial genomes, though some unbinned organisms could have the ability to fix carbon. The lack of a density shift in the bulk samples indicates that carbon fixation in the bulk soil was undetectable (Fig. S2).

Some bacteria, especially PGPB, promote plant growth through the production of hormones and other compounds. Two of the bacterial genomes we reconstructed encoded the pathway for indole-3-acetic acid production, a compound that increases plant growth and induces a variety of other physiological changes (Fig. 5). Eight of the genomes encode 1-aminocyclopropane-1-carboxylate (ACC) deaminase (Fig. 5), which prevents ACC from being converted to ethylene in the plant. However, ACC deaminase is also involved in the generation of propionate. In the Streptomyces_71_13 genome, the ACC deaminase gene is surrounded by plant carbon degradation genes such as pectin lyases and pectinesterases. We identified 18 genomes with the pathways for the production of acetoin and/or 2,3-butanediol from pyruvate (Fig. 5). These bacterially produced volatile organic compounds (VOCs) diffuse through soil and can act as growth-promoting factors and stimulate a plant systemic defense. Although these compounds increase resistance to plant pathogens, the pathway can also be involved in the anaerobic fermentation of glucose (49, 50).

Microbes can also promote plant growth through nutrient generation or mobilization. Microbially produced phytases release phosphorus from phytate, a phosphorus storage compound common in soil but inaccessible to mature plants (Fig. 5) (51). Several of the genomes encoded biosynthetic pathways to produce siderophores (Fig. S6). In addition to iron acquisition, siderophores can complex iron and other metals, thus promoting the release of phosphate from insoluble soil-associated minerals (52). We did not identify any N_2_-fixing pathways in the genomes or on the unbinned scaffolds.

10.1128/mSphere.00085-21.6 FIG S6 Biosynthetic gene clusters encoded in bacterial genome bins identified in this paper. Signaling compounds includes homoserine lactone clusters, N-acyl amino acid clusters, and butyrolactone cluster. Download FIG S6, TIF file, 2.9 MB. Copyright © 2021 Starr et al. 2021 Starr et al. https://creativecommons.org/licenses/by/4.0/ This content is distributed under the terms of the Creative Commons Attribution 4.0 International license.

### Interactions between bacteria and microeukaryotes.

Several of the bacterial genomes encoded secretion systems that are thought to be associated with bacterium-eukaryote interactions. Six genomes carry multiple genes from type III secretion systems, which are known to be important in symbiotic colonization and infection of eukaryotes (53). We do not know the intended targets of the type III secretion systems because of the diversity of possible hosts. However, we identified 58 type III effector proteins with sequence homology to known plant pathogen effector proteins (mostly from *Ralstonia*, Pseudomonas, and *Xanthomonas*) encoded on the Burkholderiales_62_29 genome (Fig. 5). We also identified 13 genomes with probable type IV secretion systems (Fig. 5), which are used in conjugation or the injection of protein-DNA complexes into eukaryotic cells (54).

Some of the assembled bacterial genomes encode systems that may influence interactions with soil fungi. Nine of the partially complete genomes encoded fusaric acid resistance proteins, which protect from the antibiotic produced by Fusarium species (Fig. 5). The Variovorax_paradoxus_68_14 genome appeared to encode three fusaric acid resistance modules; one was near an esterase and phospholipase C which hydrolyzes phosphatidylcholine, an important fungal phospholipid (55). In one Streptomyces_71_13 genome, the fusaric acid resistance module is near two glycoside hydrolases which may act on fungal cell walls. Included in this region is a ceramidase, which hydrolyzes glucosylceramides, a necessary metabolite for Fusarium pathogenesis and morphology (56). Another possible indication of bacterial-fungal relationships is bacterial genes for decomposition of fungal compounds. The Streptomyces_71_13 genome encodes 39 enzymes that target fungal biomass, 30 of which have an identifiable secretion signal, indicating they are exported extracellularly (Fig. 5).

We also identified some evidence for bacterially produced defenses against grazing. Bacteria often use extracellular polymeric substance (EPS) production, specific secondary metabolites, and active infection to deter grazing (57). EPS production is common in soil bacteria but difficult to infer from genome information. One indication of EPS formation is the production of proteinaceous adhesins (58). Many of the genomes we investigated encoded adhesins, with Verrucomicrobia_58_7, Verrucomicrobia_63_30, and Variovorax_paradoxus_68_14 genomes encoding the most (Fig. 5). Several genomes encode the pathway to produce hydrogen cyanide (HCN) (Fig. 5). Eight of the genomes encode a insecticidal toxin subunit TcC which is lethal to certain insects, and possibly nematodes (Fig. 5) (59). The Acidobacteria_68_21 genome encoded two complete Tc insecticidal toxin modules and an insecticidal crystal protein related to the bt toxin from Bacillus thuringiensis (Fig. 5).

### Bacterium-bacterium interactions.

In addition to bacterial interaction, cooperation, and competition with eukaryotes, genomes can provide indications of interbacterial interactions in soil. We identified some of the best characterized mediators of interbacterial interactions, including signaling molecules such as acyl-homoserine lactones, autoinducing peptides, indoles, gamma-butyrolactones, and a variety of other compounds (Fig. S6). In addition, many genomes encoded one or more quorum-quenching genes, which may act either to degrade self-produced quorum molecules or as a means to disrupt other bacterial species communications.

We identified a large number of biosynthetic gene clusters (BGCs) in the bacterial genomes, especially polyketide and nonribosomal peptide biosynthetic gene clusters (Fig. S6). Several *Streptomyces* spp. encoded many BGCs, and several *Burkholderiales* and *Acidobacteria* genomes also encoded a high number of secondary metabolite clusters, as has been found in other soils (12, 60). Some of the BGCs were located near other genes of interest for instance, the Streptomyces_turgidiscabies_70_12 genome encoded three BGCs near 10 plant cell wall hydrolysis proteins. Evidence for competition between closely related strains comes in the form of bacteriocins, antibiotics that act on closely related bacteria. These were found in nearly 40% of the genomes. Eleven genomes encoded type VI secretion systems, which inject effectors into neighboring bacteria (Fig. 5). Many genomes had multiple VgrG proteins, the tip of the needle and effector transporter, near large proteins with Rhs repeat domains which may function as bacterial toxins (Fig. 5) (61).

### Evidence of bacterium-phage interactions.

Many of the phage we identified appeared to be highly ^13^C labeled (Fig. 4), providing direct evidence of plant-derived C moving through trophic levels. Indeed, in the week 6 sample, the two most labeled entities were phage (Fig. 3). We focused on circularized phage genomes (as opposed to those integrated into the host genome as a prophage where they could become ^13^C labeled through host growth alone) as these are likely complete genomes and the product of active infection during our experiment.

We identified Burkholderiales_62_29 as the possible host for one of the most highly labeled phage, *Burkholderiales* phage 68_11, based on the match between a CRISPR-Cas spacer and the complete phage genome. The spacer hit the large terminase subunit with two mismatches (33 bp total). It appears the phage may be capable of a lysogenic life cycle because of the presence of a serine recombinase and a possible induction region consisting of a histone-like protein and lambda repressor-like gene.

Another phage-host connection we identified was based on a recent lateral gene transfer event. The *Catenulispora* phage 69_17 was highly ^13^C labeled and ranked as the 1st and 9th most labeled entity in the week 6 and 9 rhizosphere samples, respectively (Fig. 3). The probable host, Catenulisporales_70_18, encodes a glycoside hydrolase (GH25) which shares 77% amino acid identity with the phage protein, and a phylogenetic tree indicates that the phage and bacterial proteins are more related to one another than to other publicly available sequences (Fig. S7). This suggests the phage may have acquired the gene from this bacterial host population via lateral transfer. The acquired gene may be a lysis factor, as GH25 breaks down peptidoglycan. The phage has a lysogenic life cycle based on the presence of a gene annotated as a tyrosine recombinase and induction regulation genes.

10.1128/mSphere.00085-21.7 FIG S7 Phylogenetic tree of GH25 gene. The GH25 genes from phages are highlighted in green, and the bacterial genome bin from this study is shown in blue. Download FIG S7, TIF file, 2.8 MB. Copyright © 2021 Starr et al. 2021 Starr et al. https://creativecommons.org/licenses/by/4.0/ This content is distributed under the terms of the Creative Commons Attribution 4.0 International license.

The remaining eight complete phage genomes could not be linked to a specific host in our data set. Many carry DNA methylation genes that may protect the phage DNA from detection or destruction by host antiviral systems. Of the 55 draft bacterial genomes, only 2 contained identifiable CRISPR-Cas systems, the *Burkholderiales* described above and Acidobacteria_68_21, which encoded two Cas type III-B systems and a type I-C system (Fig. 5).

## DISCUSSION

We combined stable-isotope probing with genome-resolved metagenomics to trace the flow of plant-derived carbon into bacterial primary consumers and then to phage and bacteriovores. Through the generation of genome bins, we were able to discover clues regarding the genomic potential and ecological roles of these organisms and to develop ideas about the nature of the movement of carbon through the system. The heavy fractions yielded larger assemblies than the lighter fractions, likely due to the reduced diversity of sequenced DNA. These larger assemblies and the reduced diversity likely improved binning by providing more complete sequencing of the present bacteria.

Our approach, which identified single copy rpS3 genes from different samples and their distribution across isotopically enriched fractions, indicates that the supply of labeled plant root carbon can help to identify assemblages of active rhizosphere organisms that are distinct from the background soil community. Based on the rpS3 analysis, a large portion of organisms in the rhizosphere were not detectably responsive to the influx of plant-derived carbon and their communities were indistinguishable from bulk samples. Successional shifts with time may have occurred, but the replication (and thus resolution) of our study was not sufficient to detect them. In a parallel highly replicated experiment, sequencing of 16S rRNA genes did identify effects of sampling time during plant growth on microbial composition (41). In addition to bacteria, we identified a number of soil eukaryotes, and though we recognize that the number we identified does not scratch the surface of soil eukaryotic diversity, our assembled metagenomes provide complete 18S rRNA sequences without the primer bias inherent to tag-based methods. We also identified 10 complete phage genomes which likely represent some of the most abundant phage in our system. This is a small number compared to the total diversity of phage likely present in the soil, yet these complete genomes allow us to predict lysogenic lifestyles and to identify possible hosts of some of the dominant phage.

By assuming linear growth over the course of the experiment, we derived gross growth rate estimates for prokaryotes, microeukaryotes, and phage that rely on root-derived carbon. From our analysis, both phage and bacteria showed higher average growth rates than eukaryotes. This may be because the eukaryotes were not feeding on enriched rhizosphere bacteria, they were slow to replicate their genomes, or the process of sieving soil and constructing the microcosms decreased their population. While we acknowledge these estimated rates have associated uncertainty, as do estimates of the abundance of metagenome-assembled genomes in soil, this approach remains one of the only means to estimate population-specific growth rates *in situ*. The use of a larger number of density fractions does result in much reduced uncertainties in growth rate estimates (62). These quantitative metrics enable us to directly link previously undescribed populations of rhizosphere microbiota to plant-derived C fluxes in an intact plant-soil environment. Future development of this technique will allow us to better understand complex carbon utilization networks in soil.

Many of the most highly labeled organisms were those with probable plant interaction systems and may span the spectrum from mutualist to pathogen. By analyzing genomes with ≥70% completeness, we were able to identify genes and pathways involved in interaction but also investigate their genomic context in order to gain additional information to help predict the purpose of these genes (63, 64). For instance, in the Streptomyces_71_13 genome, the ACC deaminase gene is adjacent to plant cell wall hydrolysis genes and the biosynthetic pathway for producing ectoine, a compatible solute common in PGPB (40). This region of the genome may enable this *Streptomyces* sp. to limit plant stress responses by lowering ethylene levels and producing an osmoprotectant which would ensure greater plant growth (65, 66). The plant cell wall digestion enzymes may enable this PGPB to invade the plant to form a closer mutualistic association with the roots. Apart from that specific region of the genome, the Streptomyces_71_13 genome also encodes a number of other pathways and genes known to be important in PGPB, including production of indole-3-acetic acid and 2,3-butanediol. The possible close association with plant growth promotion may explain why Streptomyces_71_13 was the most labeled population in our samples collected at week 9.

In addition to possible PGPB we also identified probable plant pathogens in the group of ^13^C-enriched taxa. Based on the presence of a type III secretion system and plant effector proteins, Burkholderiales_62_29 may act as a plant pathogen. If so, this would have enabled the assimilation of large amounts of root-derived carbon and contributed to making this genome the 17th most labeled population in week 6. To further evaluate this hypothesis, we referred to the transcription of the effector proteins from a related study of the *Avena* rhizosphere metatranscriptome which used the Burkholderiales_62_29 genome as a reference; 8 of its 58 effectors were statistically upregulated in the rhizosphere compared to bulk soil transcription (67). In addition, we identified a labeled Fusarium sp. (SM_10888_Uncultured_Fungi) in the rhizosphere samples; several Fusarium spp. are known *Avena* pathogens and are able to obtain carbon directly through infection of plant roots (68). Bacteria with genomes that encode a large number of fungal cell wall hydrolysis genes may obtain carbon from fungal necromass or actively antagonize living fungi through digestion of their cell walls (69).

It appears that not all bacteria growing on root-derived C have identifiable genes that predict a close relationship with the plant. Burkholderiales_70_21, Leifsonia_69_10, and Sphingomonadales_67_17 were some of the most highly labeled bins, despite encoding few identifiable interaction systems. We identified at most 6 genes in these bins compared to the average number of 16 predicted interaction genes for all genome bins. It is possible some noninteracting organisms are well positioned to take advantage of the abundance of resources in the rhizosphere and grow quickly.

Several of the eukaryotes we identified were labeled with plant-derived ^13^C, including two nematodes, a rotifer and a rhizaria. Based on their phylogeny, these microeukaryotes may lead a bacterivorous lifestyle. Their ^13^C enrichment in our study indicates that they consumed rhizosphere bacteria that were actively consuming root-derived carbon. This represents a flow of root-derived carbon through two trophic levels from plant to primary consumers and into predators. Several of the labeled bacterial genomes encoded systems that may act as grazing deterrents, for instance the pathway to produce HCN which acts as a nematicidal agent (70).

In this study, we traced carbon movement though two trophic levels from plant root carbon into bacterial genomes and then into phage genomes. Stable-isotope probing enabled us to identify the most actively infectious phage in the rhizosphere. We infer that the complete phage genomes are derived from phage particles or phage in the process of replication because of their circularized genomes, rather than phage integrated into bacterial genomes. Interestingly, two of the most highly labeled phage were more highly isotopically labeled than their bacterial hosts. It is likely that recently synthesized nucleotide pools are more highly labeled that other cell structural components, and these nucleotides were shunted directly into the replicating phage genomes. The presence of highly labeled phage implies that phage predation may be a major source of bacterial death, and thus nutrient cycling, in the rhizosphere.

In the bacterial genomes we assembled, we also identified possible mediators of bacterium-bacterium communication and competition. In the labeled genomes, we identified many signaling compounds and quorum-quenching genes; although we cannot definitively verify their function, it appears communication systems could be critical for life in rhizosphere soil (71). The number and distribution of interbacterial killing systems in labeled genomes, including a possible facultative predatory bacteria *Lysobacter* (72), may indicate active competition for resources or space in the rhizosphere.

Finally, one of the more intriguing genomes we reconstructed is for an *Acidobacterium*. The Acidobacteria_68_21 genome encodes a flexible metabolism and many defensive capabilities, CRISPR-Cas systems, insecticidal proteins, and 40 BGCs. While this genome was not labeled, it was one of the most abundant bacteria in bulk soil, and these defense systems may serve to protect this dominant bacterium from grazing or parasitism.

By tracing the movement of the carbon from plant roots into the soil community, we can begin to understand rhizosphere ecology, which in turn informs us about the carbon cycle in soil. The possible interactions that we identify in rhizosphere soil have the ability to impact plant growth and shape the flow and stabilization of carbon in soil. Lysis of bacteria by phage or interbacterial killing systems may release easily metabolized compounds that could be respired and returned to the atmosphere. Also, bacteria may contribute to soil aggregate stability and carbon stabilization through the production of EPS and other types of organic matter (73). PGPB could enable the plant to fix more CO_2_, ultimately increasing the amount of carbon introduced into the soil (74). Only through a better understanding of the interdomain interactions occurring in soil can we begin to understand the functioning of soil.

### Conclusions.

We used genome-resolved metagenomics applied in the context of a SIP study to generate insights into the active members of a rhizosphere soil community. We identified organisms and genetic sequences that suggest mechanisms of potential interaction; these provide fertile topics for further exploration and verification. In the long term, understanding of soil interaction networks may provide pathways to improve plant primary production and carbon compound sequestration in soil.

## MATERIALS AND METHODS

### Plant growth and ^13^CO_2_ labeling.

To generate the samples for this study, we grew common wild oat, Avena fatua, in greenhouse microcosms packed to field bulk density with grassland soil. Briefly, surface soil (0 to 10 cm) was collected from the University of California Hopland Research and Extension Center (Hopland, CA, USA), from a field dominated by *Avena* spp. The soil is a fine-loamy, mixed, active, mesic Typic Haploxeralf (75, 76). For this experiment, three separate microcosms were constructed (see Fig. S1 in the supplemental material) and maintained at approximately 15% soil moisture; additional details of the microcosm design and plant growth conditions have been documented previously (41). One microcosm was destructively harvested after 2 weeks of acclimation; before planting, this represents our time zero sample. Since this sample had no root influence, the soil is considered a “bulk” soil. In the other two microcosms, an A. fatua seedling was planted, and the microcosms were incubated in an isotope labeling chamber supplied with 99 atom% ^13^CO_2_ at 400 μl/liter CO_2_ during the day to achieve an overall atmospheric ^13^C enrichment of 90 atom%. After 6 and 9 weeks of growth, a single microcosm was destructively harvested. Rhizosphere samples were defined as soil attached to a root after gentle shaking. Paired bulk soil samples from weeks 6 and 9 came from a root exclusion mesh bag (1 μm) which was designed to exclude roots but allow the soil inside to otherwise experience identical microcosm conditions (moisture, temperature, etc.). Rhizosphere soil was washed off roots, and bulk soil was collected from the bulk soil bags; from these soils, 0.5 g was used for DNA extraction using a phenol-chloroform procedure (41). In sum, our analyses included five separate DNA samples, a time zero bulk sample, week 6 bulk and rhizosphere samples, and week 9 bulk and rhizosphere samples.

### Density separation.

We used a CsCl density gradient centrifugation to separate each DNA sample based on density using previously described methods (77). Briefly, for each sample, 5.5 μg of DNA was added to a gradient buffer with a density of 1.735 g/ml. The solution was spun in ultracentrifuge tubes (Beckman Coulter Quick-Seal, 13 × 51 mm) in an Optima L-90K ultracentrifuge (Beckman Coulter, Brea, CA, USA) using a VTi65.2 rotor at 44,000 rpm (176,284 average relative centrifugal force [RCF_avg_]) at 20°C for 109 h with maximum acceleration and braking of the rotor to maintain the integrity of the density separations. The gradient was then separated into ∼32 fractions using a syringe pump delivering light mineral oil. Each fraction (∼144 μl) was measured for density using an AR200 digital refractometer (Reichert Inc., Depew, NY, USA), and DNA was precipitated and quantified as previously described (77). Fractions were then combined into three bins based on density and by comparison between the rhizosphere samples and the associated bulk soil (light = 1.692 to 1.737 g/ml; middle = 1.738 to 1.746 g/ml; heavy = 1.747 to 1.765 g/ml; see Fig. S2 and Table S1 in the supplemental material). The heavy bin was defined as any fraction with a density greater than the point at which the bulk sample DNA concentration reached 0 ng/μl DNA. The rhizosphere middle fractions (and bulk heavier fractions) were defined as every fraction between the point where the rhizosphere and bulk lines crossed and where the heavy fraction started (Fig. S2). For the rhizosphere samples, we sequenced all three bins; for the bulk samples, we sequenced only the light and heavier bins. Thus, from the five separate DNA samples described above, we generated 12 distinct fractionated DNA samples for sequencing.

### Sequencing.

The 12 fractionated DNA samples were sequenced at the University of California (UC) Davis Genome Center on an Illumina HiSeq 3000 (Illumina Inc., Hayward, CA, USA) with paired-end libraries prepared with the Kapa Hyper protocol and a read length of 150 bp.

### Sequence preparation and analysis.

Reads were trimmed using Sickle (https://github.com/najoshi/sickle; version 1.33) with default parameters; BBtools (https://sourceforge.net/projects/bbmap/; version 35) was used with default parameters to remove Illumina adaptors and phiX sequences. Each sample was assembled individually using IDBA-UD (-step 20, -maxk 140, -mink 40) (78). Only scaffolds larger than 1,000 bp were included in further analyses. Genes were predicted using Prodigal (79). The open reading frames (ORFs) were annotated using a combined approach. Sequence similarity searches were performed using USEARCH (version 7.0.959) (80) against UniRef100 (July 2014) (81), UniProt (June 2014) (82), and the KEGG (June 2015) (83) databases. Additional gene annotations were done using hidden Markov models (HMMs) that were constructed based on KEGG Orthologies (KO) as outlined in reference 84. Briefly, all proteins assigned to a KO were clustered using MCL (85) with inflation parameter (-I) of 1.1, based on global percent identity. Individual trusted thresholds were calculated by running HMM search of all the proteins with assigned KOs against the HMM database. Clusters were aligned using MAFFT v7 (86), and HMMs were constructed using the HMMER suite (87). Protein domain-level analysis was conducted using InterProScan (88). Carbohydrate active enzymes were identified using dbCAN2 (89, 90). Secondary metabolite clusters were found using antiSMASH 4.0 (91, 92). tRNAs were predicted using tRNAScan-SE (93). The 16S and 18S rRNA sequences were found and aligned using ssu_tree.py (https://github.com/christophertbrown/bioscripts27; version 1.0). Eukaryotic 18S rRNA genes were dereplicated and clustered at 98% nucleic acid identity representing a possible species-level designation (94, 95), aligned using SSU-ALIGN (96), and trees were generated using RAxML on CIPRES (97, 98). Genomes were binned using a combined approach. We used abawaca (https://github.com/CK7/abawaca; version 1.00), MaxBin2 (99), and MetaBAT (100), the most complete bins with the least amount of contamination (as calculated by the number of 51 single copy genes [Fig. S3]) were chosen using DAS Tool (101). Further genome curation was conducted in ggKbase (101, 102), such as the removal of taxonomically divergent scaffolds which were binned incorrectly from algorithms which do not incorporate phylogenetic information (https://ggkbase.berkeley.edu/). Bins were dereplicated to the species level based on rpS3.

The rpS3 genes were identified and dereplicated to the species level (99% nucleotide identity), and the longest scaffold was chosen using rpS3_trckr (https://github.com/AJProbst/rpS3_trckr; version 1.0). Each sample was mapped to each scaffold using Bowtie2 (--sensitive and --rfg 200,300), the reads were filtered for two mismatches, and the coverage was calculated using calculate_breadth.py (https://github.com/banfieldlab/mattolm-public-scripts/blob/master/calculate_breadth.py; version 1.0), which calculates the coverage of supplied scaffolds based on mapping files produced with Bowtie2 (103). The coverage values for the rpS3 scaffolds were normalized for total read depth from the corresponding sample. Principal coordinate analysis was conducted in the R programming environment with the vegan package (104, 105). The R script is publicly available (106). rpS3 amino acid sequences were aligned using MAFFT v7.402 (86) with the E-INS-i options on Cipres (98). Trees were generated on Cipres using RaxML with the Jones-Taylor-Thornton (JTT) protein substitution model, and figures were generated using iTOL (97, 98, 107).

Phage genomes were identified using VirSorter and manually on ggKbase (108). Phage genome completeness was checked by mapping reads, as described above, and visualizing on Geneious R9.1 (109). Complete, circularized phage genomes will have uniform read coverage across the genome and reads paired across the entire span of the scaffold without repetitive elements on the end of the scaffold which could cause long paired reads instead of a circular sequence. CRISPR spacers were found using CRISPRDetect (110).

### Enrichment, sip, and growth rate.

The coverage and relative abundance of individual soil populations were calculated based on mapping reads using Bowtie2 (--sensitive and --rfg 200,300) (103). For bacterial bins, the reads were mapped to all scaffolds in the bin. For eukaryotes, we were only able to identify individual eukaryote scaffolds with the 18S rRNA gene, but we were not able to bin genomes from the eukaryotes. For eukaryotes, reads were mapped to the whole scaffold containing the 18S rRNA gene. For phage, the reads were mapped to the complete phage genomes. Reads were filtered for two mismatches, and the coverage was calculated using calculate_breadth.py. The coverage values were normalized for total read depth from the corresponding sample.

We estimated the atom percent excess (APE) ^13^C enrichment for each taxon following the procedures detailed in Hungate et al. (37), with the following adjustments for metagenome-assembled genomes instead of 16S rRNA genes. The density of an organism’s DNA was compared between labeled (rhizosphere) and unlabeled (bulk) samples, and a model of isotope substitution in DNA was used to convert the observed change in density to isotope enrichment (36, 37). In previous qSIP experiments, amplicon sequencing of the 16S rRNA gene has been used to estimate the relative abundances of bacterial and archaeal taxa. Those relative abundances were then converted to estimates of absolute abundance by multiplying by the total number of 16S rRNA gene copies using the universal 16S rRNA primer for qPCR for each density fraction in each replicate gradient. Here, we used the relative coverage (i.e., coverage normalized for read depth) of metagenome-assembled genomes as a proxy for relative abundance and total DNA concentration in place of total 16S copies in order to calculate a metric of abundance (*y*) for each taxon (*i*) in each density fraction (*k*), for each replicate (*j*) as

$$y_{ijk}=p_{ijk}\times f_{jk}$$

where *p* is relative coverage and *f* is the total DNA concentration. We acknowledge that this metric of taxon-specific abundance (*y*) is incomplete because the total rhizosphere DNA presumably represents many more taxa beyond the metagenome-assembled genomes and eukaryotic scaffolds that we identified. The qSIP analyses were conducted separately for the week 6 and week 9 data. For each time point, we used the bulk soil data from the corresponding week as the unlabeled treatment. Because our experiment was not replicated, we were unable to calculate confidence intervals for APE ^13^C based on means and standard errors, an approach that has been used previously to estimate minimum detectable differences in isotope incorporation (37). However, in our data, the relationship between population rank and APE ^13^C value exhibited a spline function pattern (Fig. S8), suggesting there is a threshold above which ^13^C-label incorporation in the bacterial genome bins, phage genomes, and eukaryotic scaffolds was detectable above background variation in the qSIP-derived estimates of ^13^C uptake. We used breakpoint analysis to test this spline function and identify a threshold value above which ^13^C incorporation could be confidently inferred. We used the “segmented” package in R, and the segmented function to identify the breakpoint, with the Davies test for significance. We focused this analysis on the 90 populations exhibiting the lowest values of qSIP-estimated ^13^C atom percent excess (i.e., from ranks 1 to 90), thereby avoiding more enriched regions where the slope of the rank-APE relationship declined (Fig. S8B). We found that including these high-rank APE values skewed the estimate of the breakpoint to near 0 APE, whereas focusing on the data surrounding the visually obvious breakpoint of interest resulted in a more conservative estimate of the detection threshold. We identified the breakpoint threshold at rank 71.1, with a standard error of 0.6. We used this breakpoint plus three times the standard error (the upper 99.7% confidence limit of the breakpoint estimate) to identify the threshold above which ^13^C uptake could be reasonably inferred. The ^13^C APE value associated with the threshold was 2.5%. Thus, we interpreted values above that APE value as having detectably incorporated the ^13^C tracer. We note that this is somewhat lower than threshold values estimated using confidence intervals calculated from replicated measurements in a 16S rRNA gene qSIP study (5.6% for ^13^C in Hungate et al. [37]).

10.1128/mSphere.00085-21.8 FIG S8 Breakpoint analysis was used to determine the minimum detectable difference of isotope incorporation for the bacterial genome bins, phage genomes, and eukaryotic scaffolds analyzed in this study. (A) Atom fraction excess (AFE) compared to the rank of bacterial bins, phage genomes, and eukaryotic scaffolds based on AFE. (B) In the inset region—focused around the predicted breakpoint region—we calculated the breakpoint in the spline curve as an indication of the lowest enrichment where ^13^C incorporation could be confidently inferred. Download FIG S8, TIF file, 2.8 MB. Copyright © 2021 Starr et al. 2021 Starr et al. https://creativecommons.org/licenses/by/4.0/ This content is distributed under the terms of the Creative Commons Attribution 4.0 International license.

For each taxon, we also estimated the gross growth rate based on plant-derived ^13^C-enriched substrates. Because some taxa may have used nonlabeled substrates for growth, this metric does not capture all growth that may have occurred during the incubation. Accordingly, these taxon-specific estimates can be thought of as measuring the degree to which individual microbial populations in the rhizosphere grew on plant-derived carbon. We used the approach outlined by Koch et al. (36), but for ^13^C instead of ^18^O (37), and we did not estimate taxon-specific mortality rates. We used a linear growth model:

$$N_{\text{TOTAL}it}=N_{\text{TOTAL}i0}+r_{i}t$$

where *N*_TOTAL_*_it_* and *N*_TOTAL_*_i0_* represent the total (labeled plus unlabeled) abundance of taxon *i* at time *t* and time zero, respectively, and *r_i_* is the net growth rate of taxon *i* with units of nanogram of DNA gram of soil^−1 ^day^−1^. Abundances were estimated as described above (for the atom percent excess calculations). We further assumed that all carbon atoms in newly created DNA could originate from the labeled C substrates in the rhizosphere, thus we set *U *= 1.0 (36).

### Data availability.

All sequence data have been made public and can be found on NCBI using the following accession numbers: BioProject, PRJNA419965; SRA, SRX6701119 to SRX6701130; complete phage genomes, MN304815.1 to MN304824.1; eukaryotic 18S rRNA, MT533858 to MT533883, and the metagenome-assembled genome bins, JAEKKE000000000 to JAEKMG000000000. Additional data, including raw trees and genome annotations, are available on Figshare (https://doi.org/10.6084/m9.figshare.c.5405805).
