# Supplementary figures and images for: Stable-Isotope-Informed, Genome-Resolved Metagenomics Uncovers Potential Cross-Kingdom Interactions in Rhizosphere Soil
Source: mSphere. 2021 Sep 1;6(5):e00085-21. doi: 10.1128/mSphere.00085-21 (PMC8550312; doi:10.1128/mSphere.00085-21)

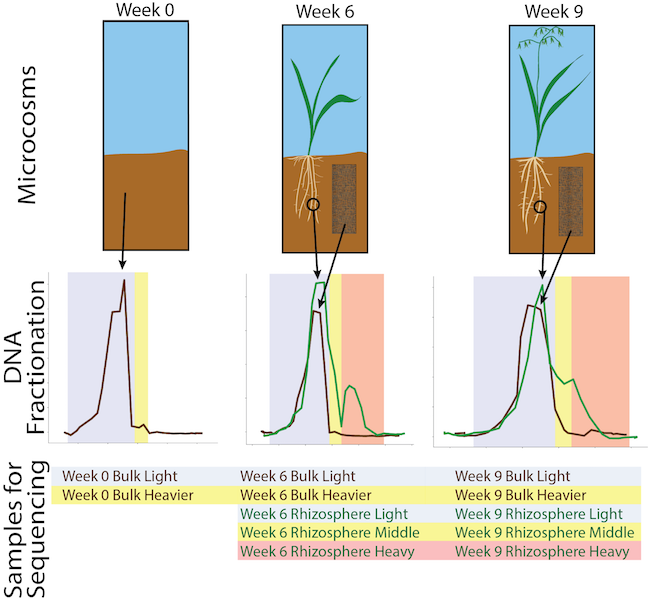

Supplement: FIG S1 [file msphere.00085-21-sf001.tif]

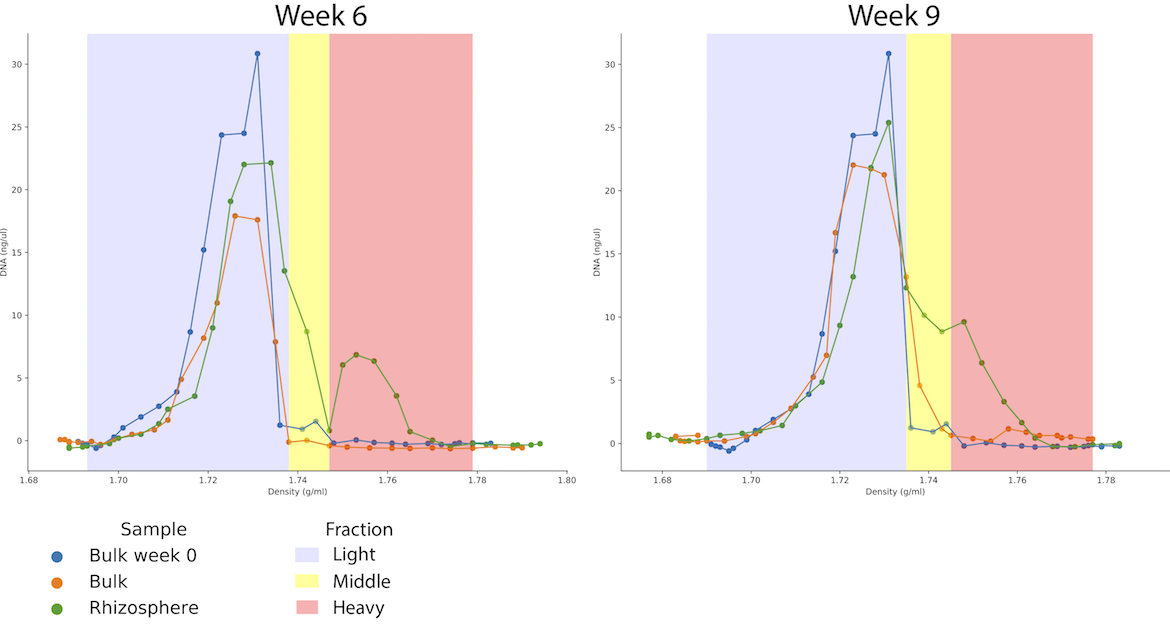

Supplement: FIG S2 [file msphere.00085-21-sf002.tif]

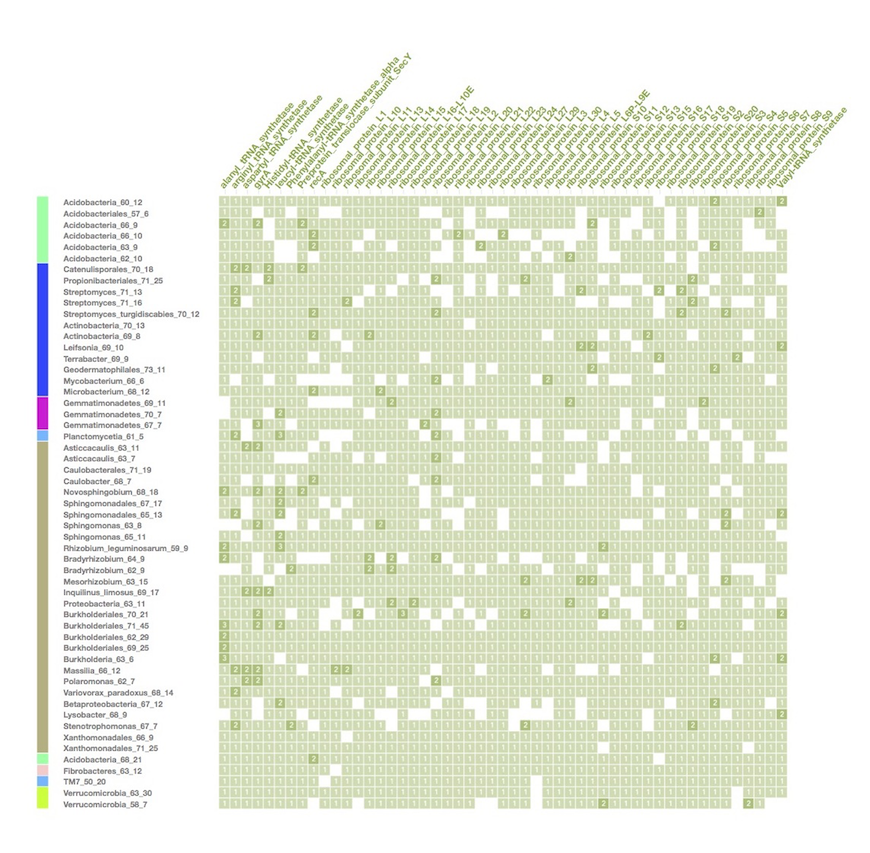

Supplement: FIG S3 [file msphere.00085-21-sf003.tif]

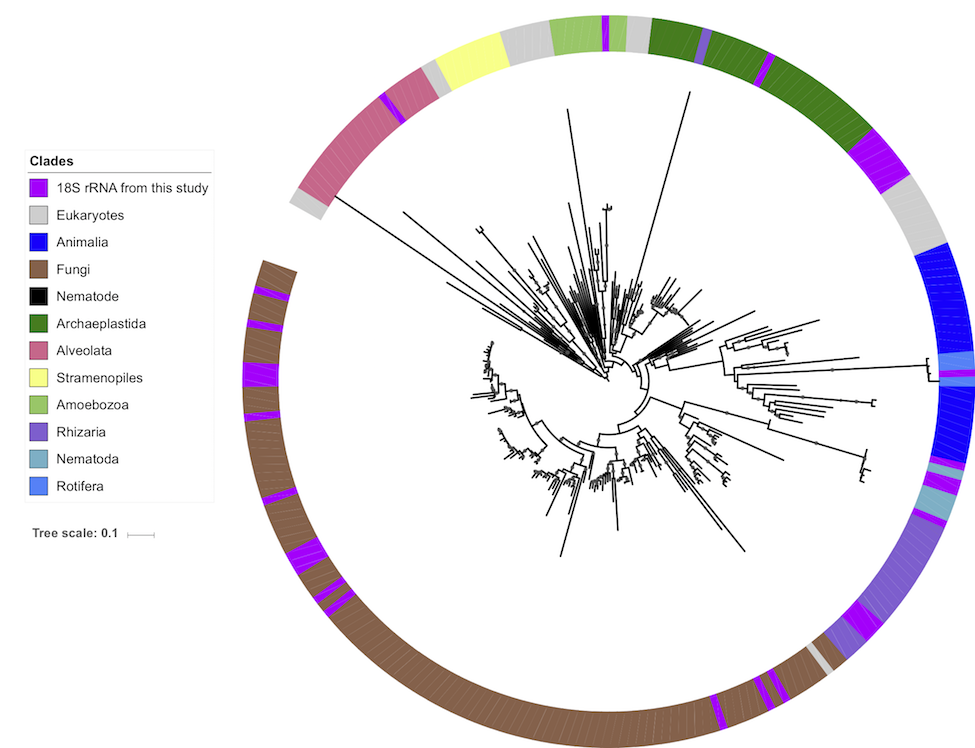

Supplement: FIG S4 [file msphere.00085-21-sf004.tif]

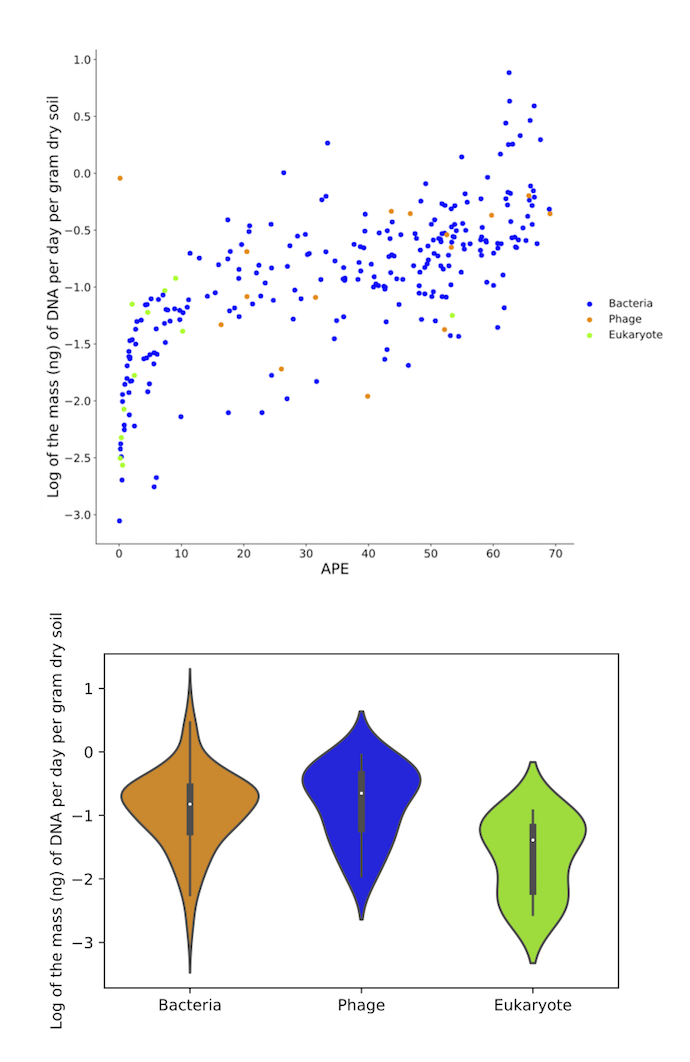

Supplement: FIG S5 [file msphere.00085-21-sf005.tif]

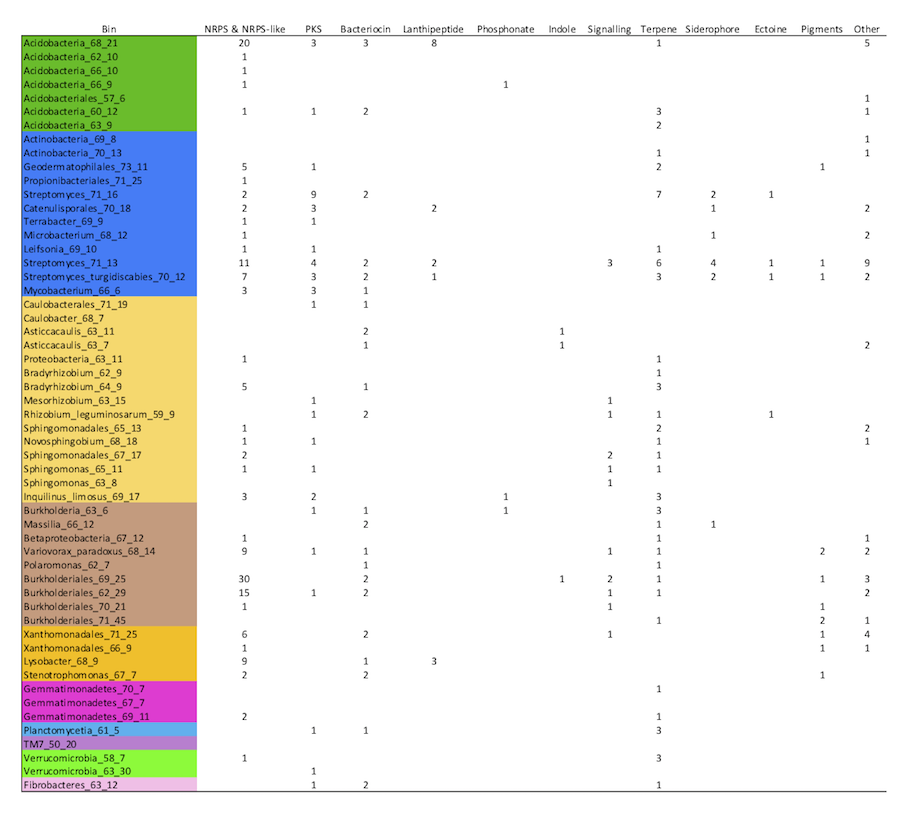

Supplement: FIG S6 [file msphere.00085-21-sf006.tif]

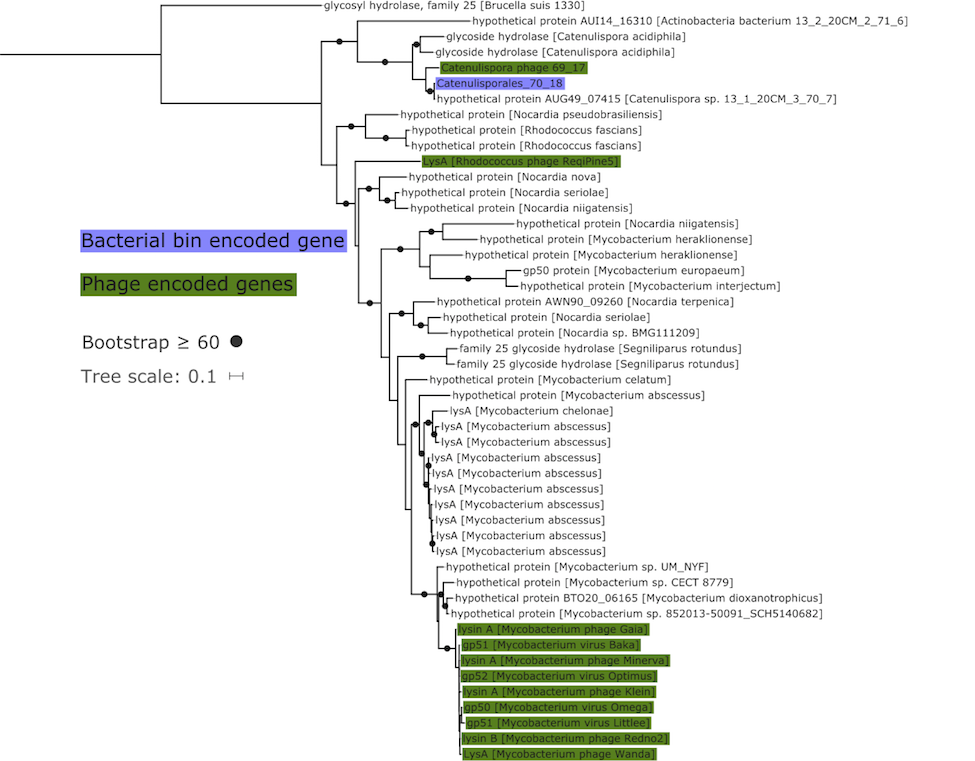

Supplement: FIG S7 [file msphere.00085-21-sf007.tif]

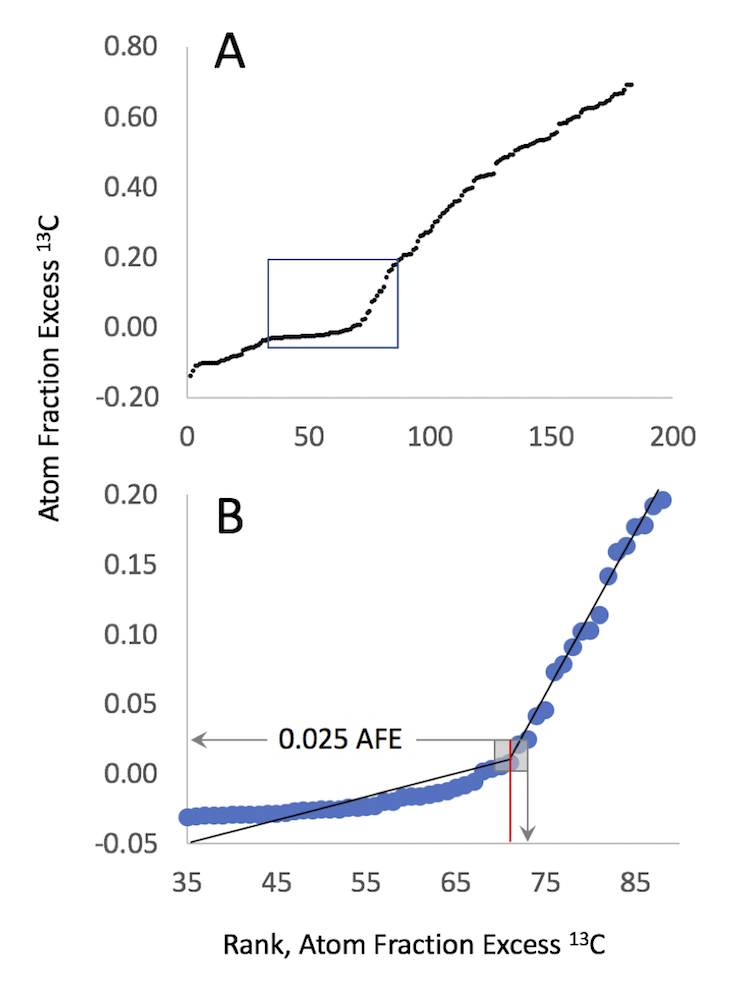

Supplement: FIG S8 [file msphere.00085-21-sf008.tif]
