# Supplementary material for: Stable-Isotope-Informed, Genome-Resolved Metagenomics Uncovers Potential Cross-Kingdom Interactions in Rhizosphere Soil
Source: mSphere. 2021 Sep 1;6(5):e00085-21. doi: 10.1128/mSphere.00085-21 (PMC8550312; doi:10.1128/mSphere.00085-21)
Supplement: TABLE S1 [file msphere.00085-21-st001.pdf]

Sample	Time point (weeks)	Fraction	Density (g/ml)	DNA concentration (ng/ul)	Total DNA (ng)	Total sequenced (Gbp)	Assembly length (Mbp)	Overall alignment rate
T0 bulk soil	0	Light	1.695-1.731	271	2519	18.4	163	11%
T0 bulk soil	0	Middle	1.732-1.744	22	176	16.8	267	24%
Bulk soil	6	Light	1.694-1.735	291	2039	17.3	97	10%
Bulk soil	6	Middle	1.736-1.742	11	109	17.4	387	30%
Rhizosphere soil	6	Light	1.692-1.737	267	2421	16.8	112	11%
Rhizosphere soil	6	Middle	1.738-1.746	17	159	15.2	211	21%
Rhizosphere soil	6	Heavy	1.747-1.765	137	1200	17.3	470	60%
Bulk soil	9	Light	1.694-1.735	113	2260	18.3	178	13%
Bulk soil	9	Middle	1.736-1.745	6	127	16.4	322	26%
Rhizosphere soil	9	Light	1.69-1.731	82	1635	18.9	176	12%
Rhizosphere soil	9	Middle	1.732-1.743	31	626	16.6	229	20%
Rhizosphere soil	9	Heavy	1.744-1.768	21	427	19.5	575	53%
